# Optimizing Gynecomastia Correction Surgery: Efficacy and Safety of Tumescent Local Anesthesia Approach

**DOI:** 10.1007/s00266-024-04404-4

**Published:** 2024-09-25

**Authors:** Matilde Tettamanzi, Edoardo Filigheddu, Federico Ziani, Giovanni Arrica, Claudia Trignano, Corrado Rubino, Emilio Trignano

**Affiliations:** 1 Department of Surgical, Microsurgical and Medical Sciences, Plastic Surgery Unit, University of Sassari, Sassari, Italy; 2 Department of Biomedical Sciences, University of Sassari, Sassari, Italy

**Keywords:** Gynecomastia surgery, Breast, Tumescent local anesthesia, Liposuction, Periareolar excision, Outpatient surgery

## Abstract

**Background:**

Tumescent local anesthesia (TLA) involves infusing a saline solution containing lidocaine and epinephrine into tissues to achieve localized anesthesia and vasoconstriction. While liposuction under general anesthesia remains the most used treatment, we introduce a novel TLA approach for gynecomastia surgery, drawing from our extensive experience in recent years.

**Methods:**

Between the years 2010 and 2023, we performed gynecomastia surgery on 60 male patients under TLA. The gynecomastia was treated by liposuction plus periareolar excision technique. Liposuction was carried out on both breasts in every case, regardless of whether the gynecomastia was bilateral or unilateral. The tumescent solution consisted of 25 mL of 2% lidocaine, 8 mEq of sodium bicarbonate, and 1 mL of epinephrine (1 mg/1 mL) in 1000 mL of 0.9% saline solution. The solution was infiltrated between the pectoral fascia and the mammary gland, and then the surgery was carried out.

**Results:**

The average volume of tumescent solution infiltrated during TLA was 300 mL per breast. There were no reports of adrenaline or lidocaine toxicity, and no cases required a conversion to general anesthesia. Patients experienced no pain or discomfort during the preoperative infiltration or surgical procedure. We observed a major postoperative complications rate of 6.7%, represented by three incident of hematoma and one case of seroma. A minor complication rate of 5% was observed: two cases of retraction of the NAC and one case of gynecomastia recurrence, the latter undergoing an additional combination procedure with liposuction and subcutaneous mastectomy. Follow-up time ranged from 30 days to 1 year.

**Conclusions:**

We developed a new outpatient surgical method for gynecomastia using liposuction and periareolar excision under tumescent local anesthesia. This technique, supported by a comprehensive rehabilitation plan, proved a successful and quick recovery, and high patient satisfaction. Our results suggest it is a feasible and effective option, warranting further consideration in gynecomastia treatment strategies.

**Level of Evidence IV:**

This journal requires that authors assign a level of evidence to each article. For a full description of these Evidence-Based Medicine ratings, please refer to the Table of Contents or the online Instructions to Authors www.springer.com/00266

## Introduction

Gynecomastia, characterized by the benign enlargement of male breast tissue, poses both physical and psychological challenges for affected individuals [1]. While the etiology of gynecomastia varies, ranging from hormonal imbalances to medication side effects, its impact on self-esteem and quality of life can be profound. Surgical correction remains a definitive treatment option, offering patients the opportunity to restore a masculine chest contour and alleviate associated [2, 3]. Depending on morphology and volume, Simon in the year 1973 classified it into four different groups. Group I encompass patients with minor breast enlargement without skin redundancy, group IIa includes patients with moderate breast enlargement without skin redundancy, while group IIb patients with moderate breast enlargement with minor skin redundancy. Finally, group III comprises gross breast enlargement with skin redundancy mimicking female breast ptosis. Simon’s classification serves as an essential framework for the clinical assessment and management of gynecomastia. It emphasizes the importance of evaluating the relative proportions of adipose tissue, glandular parenchyma, and skin redundancy, which are critical for patient selection and the formulation of precise surgical strategies. [4] Among the various surgical techniques available, the utilization of tumescent local anesthesia has gained prominence for its potential to enhance patient safety, minimize intraoperative bleeding, and expedite postoperative recovery. Tumescent anesthesia involves the infiltration of large volumes of dilute local anesthetic solution, typically containing lidocaine and epinephrine, into the subcutaneous tissue [5, 6]. This technique not only provides effective pain control but also serves as a reliable method for hydrodissection, facilitating precise dissection and minimizing surgical trauma [7]. Despite the growing adoption of tumescent local anesthesia in gynecomastia correction procedures, comprehensive scientific investigations regarding its efficacy, safety profile, and perioperative outcomes remain limited. Thus, this study aims to evaluate the outcomes of gynecomastia correction surgery performed under tumescent local anesthesia, with a focus on both short-term and long-term results. By elucidating the advantages and potential limitations of this anesthesia technique in gynecomastia surgery, this research seeks to contribute to the refinement of surgical practices, ultimately optimizing patient care and satisfaction. Through meticulous examination of surgical outcomes and patient-reported experiences, this study endeavors to provide valuable insights that can inform clinical decision-making and enhance the overall management of gynecomastia.

## Materials and Methods

Between 2010 and 2023, a total of 60 individuals underwent corrective surgery for gynecomastia. All operations took place at a certified outpatient facility. The surgical team comprised a plastic surgeon with board certification, an assistant surgeon, an operating room nurse, and a certified anesthesiologist. Patients received comprehensive information about the procedure, its indications, and potential complications, such as postoperative bleeding, scar retraction, and seroma formation. Prior to surgery, patients underwent standard preoperative tests, including blood work, cardiac evaluation, and breast ultrasound. All patients met the American Society of Anesthesiologists (ASA) criteria for either status I or II. Exclusion criteria included ASA status III or higher, as well as BMI considerations, and patients with pseudogynecomastia, whose treatment typically involves weight loss and, in some cases, liposuction, but it does not require the surgical removal of glandular tissue. Simon’s classification for gynecomastia was used for grading, including in our study patients with grade I, IIa, and IIb. Those patients with grade III gynecomastia with significant excess skin have been excluded, since this approach may be inadequate for addressing the skin redundancy, which often requires more extensive surgical techniques to achieve optimal esthetic outcomes. Medications affecting platelet function were discontinued 5–7 days before surgery or substituted with suitable alternatives. Each patient received peripheral intravenous access, and vital signs were continuously monitored throughout the surgical and recovery phases. Breast anesthesia involved the use of Rusciani’s tumescent solution, consisting of 25 mL of 2% lidocaine, 8 mEq of sodium bicarbonate, and 1 mL of epinephrine (1 mg/1 mL) in 1000 mL of 0.9% saline solution, with a total volume of 250–350 mL per breast [6]. Rusciani’s tumescent solution differs from Klein's solution because it contains lower levels of lidocaine, reducing the risk of toxicity. The cutaneous incision site was infiltrated with 1% lidocaine containing 1:100,000 epinephrine. During the anesthesia process, the plane between the gland and the superficial fascia of the pectoralis major muscle was identified, and a spinal needle connected to a peristaltic infiltration pump was placed in this plane. The infusion was halted upon reaching an average volume of 300 mL per breast. The amount of tumescent solution administered varied based on chest size and BMI, ensuring breast turgidity and minimizing the risk of drug toxicity, particularly in patients with smaller chests and glandular tissue and lower body weights. Glandular infiltration ensured thorough anesthesia through direct contact. The initial incision was made 20 minutes later to allow for the full effect of epinephrine and lidocaine. Before anesthesia administration, the patient was positioned upright for preoperative markings and photography. Markings included the inframammary fold, sternal midline, and two small incisions on each side of the chest for introducing the liposuction cannula. These incisions were made along the anterior axillary line: one at the level of the inframammary fold and another at the superior border of the breast. A 3-mm-diameter liposuction cannula with a Mercedes tip was inserted through the inframammary incision for liposuction of the ipsilateral breast, followed by crisscross liposuction from the superior incision to disrupt the inframammary fold. This process was repeated on the opposite breast using the LipoSurg device [8, 9].

Following liposuction, approximately a 5-mm-thick layer of fat was left beneath the skin across all breast areas to maintain a valid subcutaneous tissue. However, sub-areolar glandular tissue remained unaddressed at this stage. The aspirated fat volume from each side was measured, with an average of 240-500 cc aspirated per breast. A periareolar incision, utilizing the Webster technique, was made from the three to nine o’clock position in a semicircular manner, followed by glandular tissue excision using scissors and electrocautery. A small portion of gland tissue, approximately 1 cm in size, was preserved beneath the nipple–areola complex to maintain vascularity and prevent excessive scar retraction. Closure of the periareolar incision was performed in two layers: the inner subcutaneous layer with intermittent delayed absorbable suture (Monocryl 3-0) and the skin with subcuticular absorbable suture (Monocryl 4-0). The liposuction stab incisions were closed with a single stitch of Dafilon 4-0. Following closure, a sterile padded dressing was applied, and immediate application of a pressure garment ensued. Drains were not utilized. After a 4-hour observation period, patients were discharged. Depending on allergy status, an oral antibiotic regimen (amoxicillin 875 mg/clavulanic acid 125 mg or ciprofloxacin 500 mg twice daily) was prescribed for 5 days, with postoperative follow-ups scheduled at 1 day, 1 week, 2 weeks, 1–3–6 months, and 1 year.

## Results

**Fig. 1:**
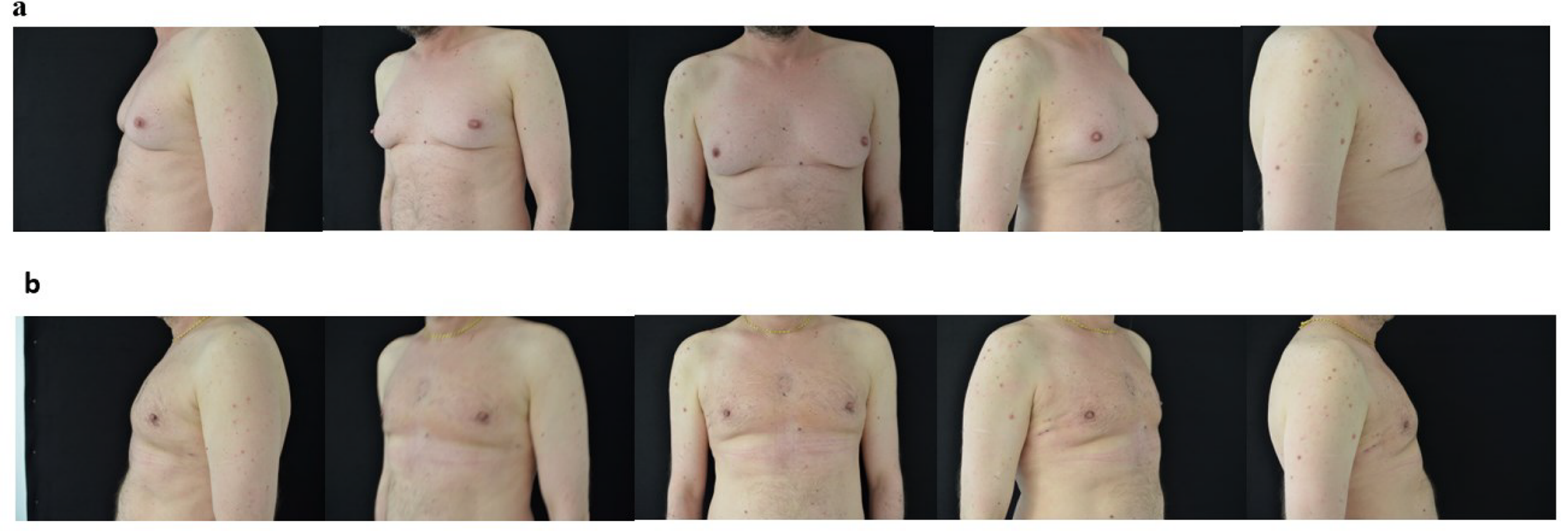
**a** Preoperative view. **b** Postoperative view after 1 year

**Fig. 2:**
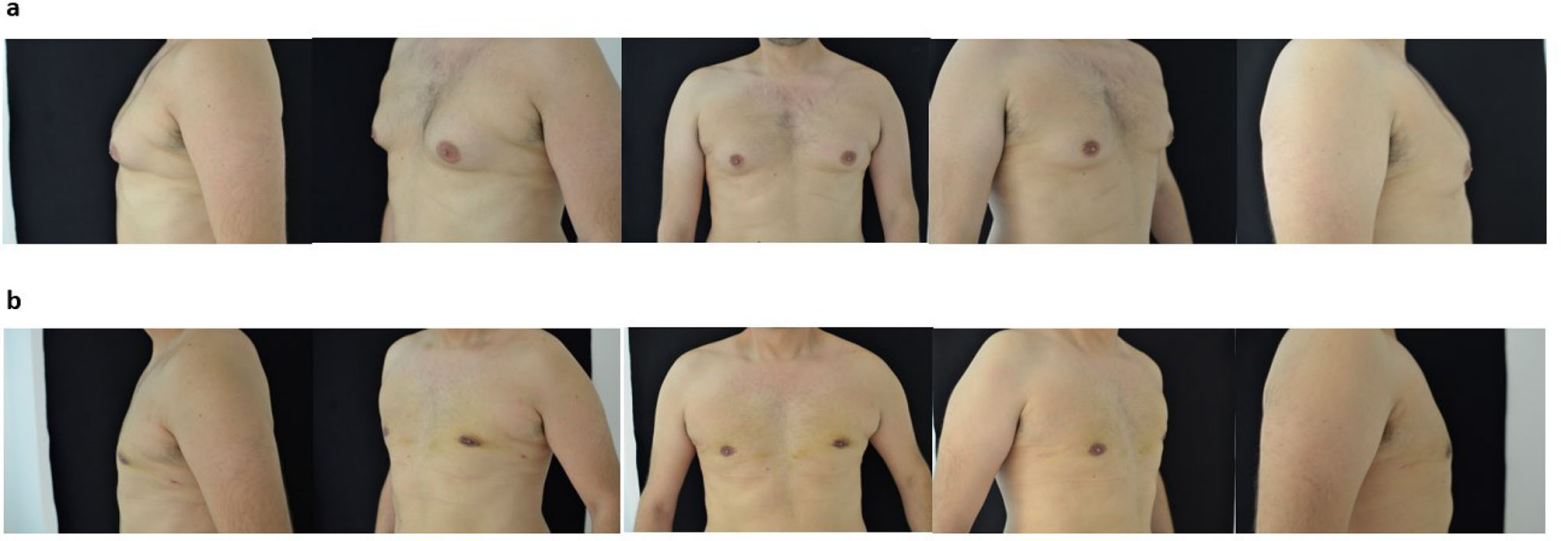
**a** Preoperative view. **b **Postoperative view after 1 year

Over the course of a 13-year interval, a comprehensive analysis was conducted on a cohort comprising 60 male individuals who had undergone gynecomastia correction procedures, exclusively employing the Tumescent Local Anesthesia technique. Most of the study subjects were between 21 and 43 years of age. The mean age of the patients was 31. Sixty percent of the subjects were classified as Simon’s group IIa, while the remaining 40% were in group IIb. No statistically significant difference between the groups was observed as per age and Simon’s grade. Among all the cases, 52 were bilateral and 8 unilateral, with the latter comprising nearly 13% of the total population. In those unilateral cases, both breasts were aspirated with liposuction, but only the gynecomastia side underwent incision and breast tissue removal. Low self-confidence was the reason for surgery in most of the cases (92%), while over half of the study’s subjects were also under emotional distress. A mean volume of 300 mL of tumescent solution was infiltrated, with a range between 250 and 350 mL, and no instances of adverse effects related to adrenaline or lidocaine toxicity were documented. General anesthesia conversion was never necessitated during any of the procedures. The average time interval from solution infiltration to the initiation of skin incision was determined to be at least 20 min; a parameter determined through collaborative assessment by the surgical team and anesthesiologist. Departing from this time threshold resulted in heightened patient discomfort, while exceeding it conferred no discernible benefits to the patient. During the surgical intervention, no reports of pain emerged during the liposuction, the skin incision or the removal of the mammary gland. The mean duration of surgery employing the TLA technique ranged from 50 min to 75 min, excluding bilateral infiltration, waiting periods, and the surgical procedure until completion. For the unilateral cases, liposuction was performed on both breasts to achieve uniformity in the result, but the subcutaneous mastectomy was only performed on the breast with excess glandular tissue. Within the scope of major postoperative complications, comprising 6,7% of cases, three incident of hematoma and one case of seroma were recorded. A minor complication rate of 5% was observed: two cases of retraction of the NAC and one case of gynecomastia recurrence, the latter undergoing an additional combination procedure with liposuction and subcutaneous mastectomy (Table 1). Patients reported overall satisfaction with the TLA procedure, indicating no discomfort during the preoperative infiltration or throughout the entire surgery. Most patients were pleased with the esthetic results at the 1-year follow-up. A satisfaction survey conducted 3 months after the surgery allowed patients to rate their pain management and esthetic outcomes on a scale from "unsatisfactory" to "excellent." Most of the patients expressed high levels of satisfaction. Importantly, all patients were subject to a follow-up period exceeding one year (Figs. 1, 2).

**Table 1:** Major and minor complications

Complications	Patients	Percentage (%)
Hematoma	3	5
Seroma	1	1.7
Recurrence	1	1.7
NAC retraction	2	3.5

## Discussion

When starting therapy for gynecomastia, it is crucial to perform a thorough assessment of the patient’s medical history and rule out other potential causes, such as endocrine disorders or certain medications. It is also important to note that gynecomastia often resolves on its own and may regress spontaneously. Pharmacological treatments that address hormonal imbalances can be effective, especially in the early stages. However, surgical intervention is considered the most effective treatment for gynecomastia that persists beyond two years of therapy [10, 11]. Most individuals seeking surgical treatment for gynecomastia are usually young or middle-aged. They often prefer quick recovery and minimally invasive procedures due to their busy work or study schedules. Our study indicates that the surgical approach we propose effectively meets the needs of patients seeking swift recovery with minimal complications. In our study, most participants were between 21 and 43 years old, with a mean age of 31. According to our data, 87% of the patients had bilateral gynecomastia, and 13% unilateral. These findings are consistent with Tolba et al. study [12], which reported 87% bilateral gynecomastia with an average age of 22.5 years. Murali et al. [13] found over 80% bilateral cases with an average age of 33 years, and Chang Li et al. [14] reported 87.8% bilateral cases with a mean age of 27 years. The literature and our study suggest the third decade is the most common age for presentation, with bilateral pathology being more prevalent [15–17].

In terms of anesthesia, while tumescent local anesthesia alone has proved effective in many cases, we acknowledge that some patients, particularly those who are anxious or require more extensive skin remodeling, may benefit from sedation combined with TLA. Sedation could provide additional comfort and anxiety relief, making it a viable alternative to general anesthesia. This approach could offer the advantages of TLA—such as shorter recovery times and reduced complications—while also addressing the needs of patients who may find the procedure under local anesthesia alone to be stressful. Tumescent anesthesia is generally suitable even for large cutaneous excisions, although we have not encountered such cases in our practice. This technique is particularly recommended for patients who refuse general anesthesia or for those in whom general anesthesia is contraindicated, such as patients with myasthenia gravis.

Gynecomastia surgery, while generally safe and effective, is not without potential complications. Common risks include infection, hematoma, and seroma, which can occur postoperatively. Patients may also experience changes in nipple sensation, scarring, asymmetry, or irregular contours. More severe complications, although rare, can include necrosis of the nipple–areola complex and deep vein thrombosis. Understanding these risks is crucial for patients considering the procedure, as it underscores the importance of choosing an experienced surgeon and adhering to postoperative care guidelines to minimize potential complications. In our study, the overall complication rate was 11.7%, including both major and minor complications. Specifically, the rate of major complications (hematoma and seroma) was 6.7%, while minor complications (recurrence and NAC retraction) accounted for 5%. These findings align with existing literature on gynecomastia correction surgery using the TLA procedure. In a recent study by He et al. [18], a fast-track approach combining tumescent anesthesia with liposuction and the pull-through technique was employed, resulting in a 5% complication rate, including seroma and superficial nipple necrosis. Another study by Mohan et al. [19] used tumescent anesthesia with periareolar excision, reporting a 6% complication rate, which included recurrences, seromas, and hematomas. Tripathy et al. [20] compared the outcomes of 20 patients treated with general anesthesia plus tumescent solution using either the pull-through or periareolar excision techniques. No complications were observed in the pull-through group, while two cases (10%) of hematoma were noted in the periareolar excision group. Boni [21] reported no early postoperative complications with TLA-powered liposuction for treating enlarged male breasts, although this method is primarily effective for pseudogynecomastia. Tumescent liposuction, initially described by Klein [22], was combined with periareolar excision in our approach for several reasons. This technique is simple and effective, eliminates the need for drainage due to minimal tissue injury and careful hemostasis, and reduces postoperative complications. Additionally, the absence of drains significantly decreased recovery time, allowing patients to resume normal activities sooner [23, 24].

The procedure, performed under local tumescent anesthesia, enabled patients to go home immediately after surgery. Regarding lidocaine toxicity, our study used approximately 300 mL of tumescent solution per breast, well within the safety limits of 55 mg/kg for adults [25, 26]. Lidocaine toxicity is linked to plasma levels influenced by rapid absorption, impaired liver metabolism, or drug interactions [27]. Therefore, patient monitoring during infiltration is crucial for safety. Moreover, avoiding propofol, ketamine, or fentanyl reduces risks like respiratory depression and blood pressure fluctuations. The brief recovery time offsets the longer preparation time required for local anesthesia infiltration. Tumescent local anesthesia, including epinephrine, causes vasoconstriction, reducing blood loss during surgery [28]. Anyways, we recommend having an anesthesiologist present to monitor oxygen saturation and assess respiratory and cardiovascular status [29], with the option to convert to general anesthesia, if necessary, though we have not encountered this need. Indeed, candidates should meet ASA status I or II criteria [30, 31].

Our study demonstrates the feasibility and safety of TLA, highlighting its potential to minimize complications and reduce patient discomfort. Traditionally, gynecomastia surgery has used general anesthesia, resulting in longer recovery periods [32].

We opted for local tumescent anesthesia, which minimizes the stress response, eliminates the need for preoperative fasting, and allows postoperative oral intake, reducing adverse effects and discomfort. This approach also reduces costs by eliminating general anesthesia fees. No toxicity-related incidents were observed with the tumescent solution, emphasizing its viability as a preferable alternative. We have been able to use TLA on myasthenic patients, for whom general anesthesia usually carries higher complication rates [33]. In our experience, 92% of patients sought surgery due to body image dissatisfaction and low self-confidence, often accompanied by emotional stress. This aligns with findings by Tolba and Nasr [12] and Murali et al. [13], where low self-esteem and cosmetic enhancement were significant motivators. Ridha et al. [34] also found 80% of patients pursued surgery due to low self-confidence and emotional distress. Multiple studies [13, 15, 17, 34] have shown that a combined approach of gland excision and fat suction is effective for small to moderate breast enlargements, including cases with excess skin, and in fact our study noted high patient satisfaction with surgical outcomes. Tolba M et al. [12] reported 93% patient satisfaction with liposuction and periareolar excision, while Yordanov V et al. [35] and Boljanovic et al. [36] observed similar positive results. However, the study's small sample size may limit the generalizability of the results. The reliance on questionnaires and self-reported esthetic outcomes introduces potential bias and subjectivity. Future research with larger, more diverse groups and objective measurements is recommended to enhance the findings' robustness. Emphasizing informed decision-making, thorough research, and consultation with experienced healthcare professionals is crucial for a comprehensive and supportive approach to gynecomastia interventions.

## Conclusion

We discussed our results with a surgical approach for treating gynecomastia, which involves a comprehensive rehabilitation regimen combined with the liposuction plus periareolar excision technique, all performed on an outpatient basis under local tumescent anesthesia. Our findings validate the feasibility of this proposal for gynecomastia treatment, resulting in expedited recovery and high patient satisfaction rates without an increase in complication rates. Consequently, the adoption of this approach, incorporating local anesthetic techniques, merits serious consideration in gynecomastia management strategies.
