# Correction: Optimizing Gynecomastia Correction Surgery: Efficacy and Safety of Tumescent Local Anesthesia Approach

**DOI:** 10.1007/s00266-024-04511-2

**Published:** 2024-11-12

**Authors:** Matilde Tettamanzi, Edoardo Filigheddu, Federico Ziani, Giovanni Arrica, Claudia Trignano, Corrado Rubino, Emilio Trignano

**Affiliations:** 1 Department of Surgical, Microsurgical and Medical Sciences, Plastic Surgery Unit, University of Sassari, Sassari, Italy; 2 Department of Biomedical Sciences, University of Sassari, Sassari, Italy

**Correction to: Aesthetic Plastic Surgery** 10.1007/s00266-024-04404-4

The original online version of this article was revised to correct the presentation of the authors’ names.

The original article has been corrected.

